# Electro-tuned catalysts: voltage-controlled activity selection of bimetallic exsolution particles†

**DOI:** 10.1039/d4ta00989d

**Published:** 2024-07-03

**Authors:** Harald Summerer, Kirsten Rath, Andreas Nenning, Thomas Schachinger, Michael Stöger-Pollach, Christoph Rameshan, Alexander K. Opitz

**Affiliations:** a Institute of Chemical Technologies and Analytics, TU Wien Getreidemarkt 9/164-EC 1060 Vienna Austria harald.summerer@tuwien.ac.at; b Institute of Materials Chemistry, TU Wien Getreidemarkt 9/165-PC 1060 Vienna Austria; c Institute of Solid State Physics, TU Wien 1040 Vienna Austria; d University Service Centre for Transmission Electron Microscopy, TU Wien 1040 Vienna Austria; e Chair of Physical Chemistry, Montanuniversity Leoben Leoben 8700 Austria

## Abstract

In this work, we show how the activity states of bimetallic Ni^0^–Fe^0^ catalysts exsolved from Nd_0.6_Ca_0.4_Fe_0.97_Ni_0.03_O_3−*δ*_ (NCFNi) can be influenced electrochemically. The NCFNi parent oxide was employed in the form of thin film mixed conducting model electrodes, which were operated in a humid hydrogen atmosphere. By precisely controlling the oxygen chemical potential in the parent oxide electrode *via* applying an electrochemical polarisation, we managed to selectively exsolve Ni nanoparticles from the perovskite lattice and study their catalytic activity switching characteristics. To be able to track the surface chemical changes during the switching process, electrochemical polarisation experiments were combined with near ambient pressure X-ray photoelectron spectroscopy (NAP-XPS) measurements. This *in situ* analytical approach allowed relating the difference we observed in the switching behaviour of Ni particles here and of Fe particles in a recent study, to a different kinetic interplay between electrochemical driving force and atmosphere. We propose that slow oxygen transport through nickel oxide, located at the particle/perovskite interface, is mainly responsible for the observed difference to iron exsolutions, which becomes especially evident for larger nickel particles. In addition, in the case of bimetallic exsolutions and with applied bias voltage as a control parameter, we are able to reversibly switch between three different activity states, namely bimetallic Ni^0^–Fe^0^ (medium activity), pure Ni^0^ (high activity), and the inactive oxides.

## Abstract 2

Controlling the surface state of bimetallic exsolution nanoparticles to either enhance or inhibit the reaction kinetics of H_2_ oxidation/H_2_O splitting by applying a voltage.

## Graphical abstract

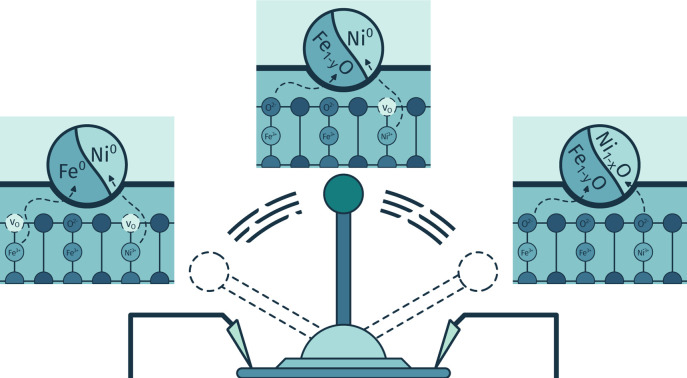

## 1 Introduction

Catalysts are an integral and indispensable part of the chemical industry and play an essential role in the production of about 80% of all manufactured goods.^1^ Their areas of application are manifold: reduction of energy consumption for a specific reaction, enabling previously impossible reaction paths, and prevention or minimisation of undesirable or toxic by-products.^2^ Due to these aspects, catalysts are also an enormously important tool with regard to the transformation of our society and industry towards a sustainable supply with energy, raw materials and everyday goods. To reach such a sustainable future, continuous development of available catalysts is necessary, especially with regard to activity, selectivity and efficiency.^3^ The use of nanomaterials often provides an effective means to improve the properties of existing catalysts or to obtain completely new types of catalysts with unprecedented properties. While many nanostructured catalysts are already in use today in the form of metal nanoparticles sitting on a ceramic support, conventional manufacturing processes (*e.g.* infiltration,^4–6^ co-precipitation^7^ or wet impregnation^8^) suffer from particle agglomeration issues and hence offer little control over particle size, density and long-term stability.

In the past decade, novel so-called exsolution catalysts have emerged and attracted rapidly growing interest, since they show great promise to circumvent these issues. In contrast to conventional oxide supported metal nanoparticles, exsolution catalysts employ a perovskite-type (ABO_3_) parent oxide which contains the desired catalytically active transition metals on the B-site – either as a dopant or a structural element. Upon reduction at elevated temperatures, the transition metals exsolve from the parent lattice and form strongly anchored, highly active and well-dispersed metallic nanoparticles on the oxide surface. Furthermore, the high redox cycling resistance of such exsolved particles offers an excellent way to get rid of catalyst poisoning or inactivation effects. Exsolution catalysts can be realised with transition metals (*e.g.* Fe,^9–14^ Co,^15–19^ Ni,^20–26^ Cu,^17,18^ Mn^16^) but also platinum group metals (*e.g.* Pt, Rh, Pd)^27–30^ or even bimetallic variants (*e.g.* Fe–Ni,^31–33^ Fe–Co,^34,35^ Ni–Co^36,37^). Especially the latter can help to further tune selectivity, activity or durability through modification of electronic and structural factors.^38^

This combination of properties proves to be game-changing in a number of fields, such as heterogeneous catalysis^29,39,40^ or solid oxide fuel and electrolysis cells (SOFC/SOEC). SOFCs/SOECs are electrochemical devices that are able to store excess electrical energy as chemical energy (SOEC mode^41–44^) or perform the reverse process and generate electricity from chemical sources (SOFC mode^45–48^). While current state-of-the-art fuel electrodes – Ni/ZrO_2_ or Ni/CeO_2_ based composites – suffer from coking issues,^49,50^ low sulfur tolerance,^51–54^ and agglomeration tendencies of the Ni phase at elevated temperatures,^55–57^ exsolution catalysts may offer a possible alternative that allows circumventing these disadvantages.

Employing exsolution-modified electrodes in high temperature electrochemical cells is not only interesting from a performance optimisation point of view, but it can furthermore allow to control the exsolution process as well as the particle redox behaviour by an additional parameter – the applied voltage. Like a thermal treatment in strongly reducing conditions,^58^ a cathodic polarisation of a mixed conducting electrode in a solid oxide electrochemical cell also reduces the parent oxide.^59^ Generally, the applied voltage across the cell affects the oxygen chemical potential (*i.e.* the effective oxygen partial pressure) in each electrode, with cathodic voltage causing low and anodic voltage high effective *p*_O_2__. Thus, a sufficiently strong cathodic polarisation of a perovskite-type electrode can mitigate the need of strongly reducing atmospheric conditions.^60^ In addition, once the particles are exsolved, the associated occurrence of higher catalytic activity can be reversed by changing the direction of the bias voltage to anodic and thus more oxidising conditions. The related increase in effective *p*_O_2__ in the parent oxide can cause an oxidation of the metal particles, going hand in hand with a decrease in catalytic activity. This ability to control the oxidation state of exsolved particles and thus their electro-catalytic activity *via* electrochemical means is known as ‘electrochemical switching’ and has already been demonstrated on several materials.^12,17,61–65^

The study of the kinetic properties of such a working electrode decorated with catalytically active nanoparticles is best done on a technologically relevant reaction, such as hydrogen oxidation/water electrolysis. This reaction is given in eqn (1) for the specific case of an iron-based perovskite-type parent oxide (that can be decorated by exsolving Fe^0^ nanoparticles):1
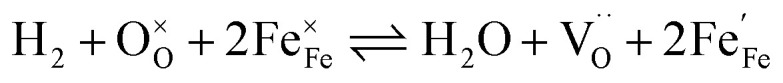


In Kröger–Vink notation eqn (1) describes the oxidation of gaseous H_2_ with lattice oxide ions 
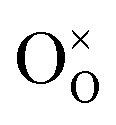
 and regular lattice iron 
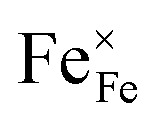
 (*i.e.* Fe^3+^) to gaseous H_2_O, reduced iron 
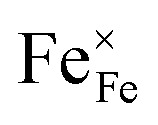
 (*i.e.* Fe^2+^) and oxygen vacancies 
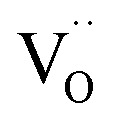
. A previous study with a La_0.6_Sr_0.4_FeO_3*δ*_ (LSF) working electrode has demonstrated that there are different reaction pathways depending on whether the exsolved particles are in a reduced state or not: the presence of Fe^0^ nanoparticles enables a kinetically fast bypass of the rate determining step on the oxide surface (which is oxidative H_2_ adsorption) by allowing dissociative H_2_ adsorption on the metallic nanoparticles, followed by a spillover of the adsorbed hydrogen species onto the perovskite surface.^12^ In a follow-up study, we could show that the redox state of the Fe^0^ particles on LSF electrodes follows the oxygen chemical potential in the perovskite electrode bulk while the atmospheric composition plays only a minor role.^65^ These results led to the question whether this dominance of the parent oxide is a general property of exsolution catalysts or whether their switching behaviour also depends on the chemical nature of the particles.

In this contribution, we are answering this question by studying the electrochemical switching behaviour of perovskite-type model electrodes with multiple exsolvable elements (in particular Fe and Ni) and their effects on the H_2_ oxidation/H_2_O splitting kinetics. For this means, *I*–*V* measurements are combined with surface sensitive near ambient pressure X-ray photoelectron spectroscopy (NAP-XPS) and scanning electron microscopy (SEM) as well as transmission electron microscopy (TEM). While research on bimetallic exsolution catalysts is still in its infancy, we address how the addition of another metal impacts the exsolution and electrochemical switching behaviour. Furthermore, we demonstrate how an applied electrochemical voltage as a control parameter also allows selective redox state modification of both transition metals independently of each other.

## 2 Experimental

### 2.1 Sample preparation

Yttria stabilised zirconia (YSZ) single crystals (5 mm × 5 mm × 0.5 mm in size, (100) oriented, mirror polished on one side, purchased from Crystec) were selected as electrolyte substrates. A triple-layered platinum/gadolinia doped ceria (GDC) counter electrode^66,67^ was prepared prior to the working electrode. To do so, a slurry consisting of Ce_0.9_Gd_0.1_O_2−*δ*_ powder (*d*_50_ = 250 nm; Treibacher, Austria) and a Terpineol based ink vehicle (www.fuelcellmaterials.com) was spincoated onto the unpolished side of the single crystal building a thin porous layer. After drying, a second, thicker layer of a custom made Pt/GDC paste was brushed on top. Lastly, a thinner layer of Pt (Tanaka, Japan) was applied. The dried sample was then sintered in air at 1150 °C for 3 h to obtain a 3D porous counter electrode. This leads to electrodes with exceptionally low polarisation resistance in reducing atmospheres^66^ and thus an excellent choice of counter electrode for our experiments.

**Fig. 1:**
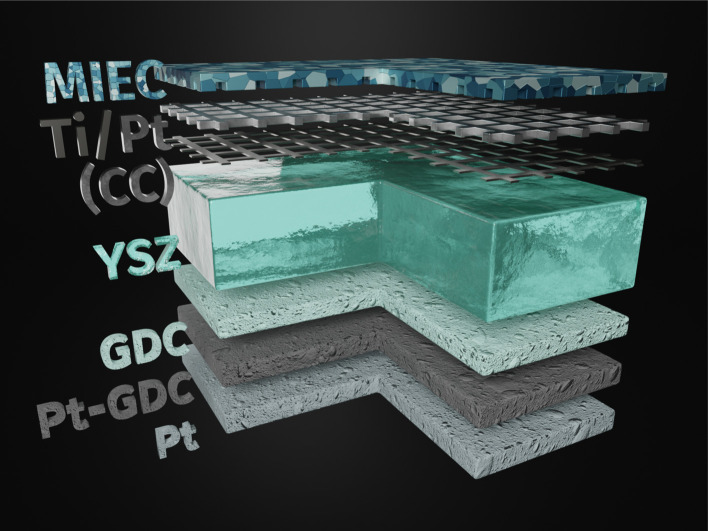
3D model of the sample: YSZ single crystal electrolyte with a working electrode consisting of a Ti/Pt current collector grid buried under a mixed conducting thin film of Nd_0.6_Ca_0.4_Fe_0.97_Ni_0.03_O_3−*δ*_ (NCFNi) on the polished top side and a triple layer counter electrode (GDC – spin-coated, Pt-GDC – brushed, Pt – brushed) on the bottom.

Before growing the working electrode, a 5 nm Ti adhesion layer and a 100 nm Pt thin film were deposited on the polished side of the single crystal *via* magnetron sputtering (BAL-TEC MED 020). By subsequent photolithography and ion beam etching the metal film was shaped into a current collector grid with 15/5 μm mesh/strip width. A thin, dense film of the actual mixed ionic electronic conducting (MIEC) working electrode material of Nd_0.6_Ca_0.4_Fe_0.97_Ni_0.03_O_3−*δ*_ (NCFNi) was then grown on top of the current collector by pulsed laser deposition (PLD). A 3D model of the sample is shown in Fig. 1.

The PLD target was prepared *via* a modified Pechini synthesis. Appropriate amounts of the precursors CaCO_3_, Fe (both Merck, 99.995%), Nd_2_O_3_ and NiO (both Alfa Aesar, 99.995%) were dissolved in nitric acid. After the addition of citric acid (Honeywell Fluka, ≥99.9998% pure, trace metals basis) in a molar ratio of 1.2 with respect to the total moles of cations, the solution was heated leading to water evaporation and subsequent self-ignition and combustion of the formed foam. Following the calcination process in air at 850 °C for 3 h, the powder was pressed uni-axially (150 MPa) into a pellet and sintered in air at 1250 °C for 12 h with heating and cooling rates of 5 °C min^−1^. Phase purity was confirmed by X-ray diffraction (XRD, see ESI† for a XRD pattern). The target was then ablated by a KrF excimer laser (Complex Pro 201F, 248 nm) at 600 °C and an oxygen background pressure of 0.04 mbar with target to substrate distance of 6 cm. The thin film thickness of 200 nm was achieved by setting the laser fluence on the target to 1.3 J cm^−2^ and firing 18 000 pulses at 10 Hz.

### 2.2 *Ex situ* electrochemical experiments

**Fig. 2:**
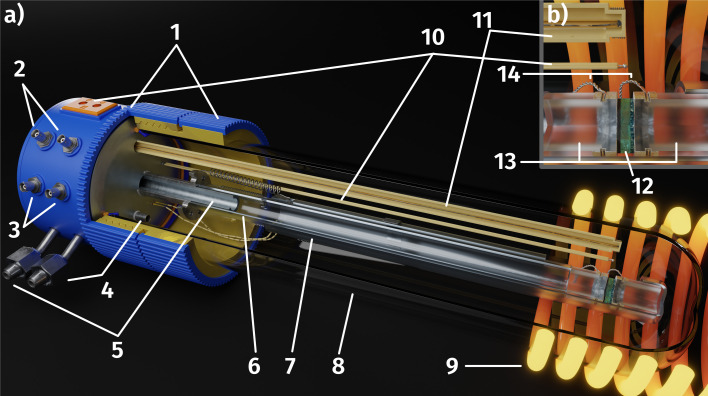
(a) Three-quarter section of the *ex situ* measurement setup: 3D printed plastic base and seal (1), BNC connections for impedance analyser or DC source meter (2) and lambda probe (3), gas outlets (4) and inlets (5), fixed inner (6) and spring loaded middle tube (7) consisting of quartz glass, sealed outer tube (8), heating coils of the tube furnace (9), thermocouple (10), custom made lambda probe consisting of a hollow YSZ pipe with Pt contacts at sample height (11); (b) magnification of hot end showing the sample (12) clamped between two silica spacer (13) covered in Pt foil with outgoing Pt wires (14).

A 3D model of the setup for electrochemical *ex situ* measurements is depicted in Fig. 2. It consists of three quartz tubes, where the inner tube was fixed and simultaneously acted as the inlet for fresh gas while the spring-loaded middle tube held the sample in place. To ensure good electrical contact and gas supply, the sample was placed directly between two silica spacers that were covered with Pt sheets and mesh. A type-S thermocouple was placed at sample height for accurate temperature readings. The outer tube sealed the complete setup, which was subsequently placed inside a tube furnace and heated to 600(1) °C sample temperature. Various mixtures of H_2_/H_2_O/balance Ar with nominal mixing rations ranging from H_2_ : H_2_O = 50 : 1 to 1 : 50 controlled by a mass flow controller system were established as measurement atmospheres. The actual oxygen partial pressure 
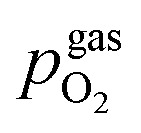
 inside the setup was determined by a lambda probe – custom made for our setup by Huber Scientifc (www.sofc.at) – which was mounted at sample height. This lambda probe consists of a YSZ pipe closed on one end with porous Pt electrodes on both gas sides of the pipe. Thus, 
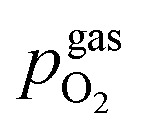
 was obtained *via* Nernst's equation with ambient air (that is present at the inner part of the pipe) as reference:2
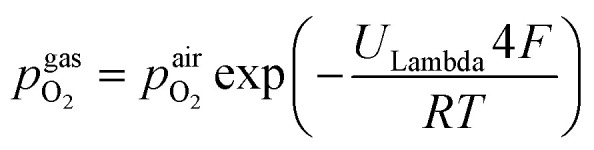


In eqn (2), 
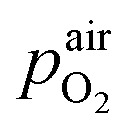
 denotes the oxygen partial pressure in ambient air (209 mbar), *U*_Lambda_ the resulting voltage, *F* Faraday's constant of 96 485.34 C mol^−1^, *R* the universal gas constant of 8.314 J K^−1^ mol^−1^ and *T* the operating temperature (873.15 K). That our lambda sensor indeed behaves according to the Nernst equation has already been confirmed in an earlier work.^65^

The aforementioned Pt sheets that contact the sample, were connected to electrical feedthroughs at the cold end of the setup *via* thin Pt wires. Electrochemical impedance measurements (EIS) were performed employing a N4L PSM 1735 Frequency Response Analyser with a FEMTO impedance converter. Impedance spectra were recorded with an AC voltage (root mean square) of 20 mV in a frequency range from 1 MHz to 100 mHz before and after each DC measurement block to obtain the YSZ resistance and confirm the stability of the working electrode (see ESI† for exemplary impedance spectra).

For DC measurements, a Keithley 2611A Source Meter Unit was connected to the sample, and two different types of experiments were performed. The first goal was to exsolve only one metal – *i.e.* the more noble Ni – by applying an appropriate bias voltage to keep the effective *p*_O_2__ in the working electrode sufficiently oxidising thus preventing the less noble Fe from exsolving. The electrochemical switching behaviour of the so obtained pure Ni exsolutions is then studied in atmospheres with varying H_2_ : H_2_O ratio, similar to our previous study on Fe exsolutions.^65^ Second, we adjusted the bias voltage so that both metals exsolve, and subsequently characterised the bimetallic exsolutions.

The first experiment required an appropriate ‘protection voltage’ to prevent co-exsolution during purging time until measurement start. This ‘protection voltage’ keeps the effective *p*_O_2__ in the perovskite electrode sufficiently high, to counterbalance the reducing atmosphere, thus only allowing the easier reducible Ni to exsolve (further details on this are given in Section 3). Then, an *I*–*V* curve was recorded by a stepwise anodic increase of 10 mV and a holding time of 120 s per step well past the expected thermodynamic potential of Ni oxidation, visible as a step change in the *I*–*V* curve. For achieving our second goal, we applied a more cathodic potential, well past the thermodynamic switching potential of the second metal (Fe). Subsequently, we checked for changes in the resulting *I*–*V* curve utilising the same recording parameters as described for the first experiment.

### 2.3 *In situ* NAP-XPS and electrochemical measurements

**Fig. 3:**
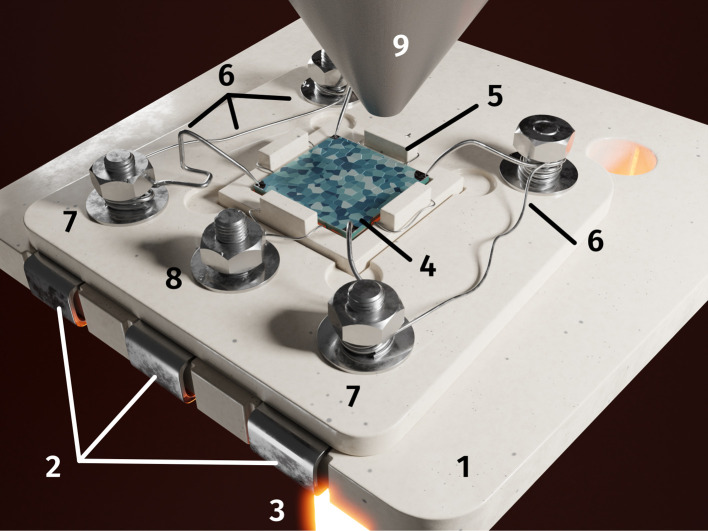
*In situ* sample holder: (1) CNC machined alumina base with (2) Pt contact pads for each electrode and a hole to allow (3) laser heating in the middle. The (4) sample is put in an (5) alumina inset with thin Pt wires (acting as contacts to the counter electrode; CE) underneath and held in place by (6) Pt–Ir needles (also acting as contacts to the working electrode; WE). Both lead to respective stainless steel screws ((7) WE and (8) CE) which in turn connect the electrodes to their respective Pt contact pads. The (9) NAP-XPS nozzle is put close to the sample for surface sensitive measurements.

In order to combine NAP-XPS and electrochemical measurements in a lab environment, a novel sample-stage was used, which was recently designed and built in-house.^68^ The sample was placed in the alumina inset with thin Pt wires underneath and a cavity in the center of a ceramic sample holder. It was contacted and held in place by Pt/Ir wires (see Fig. 3). The wires extended to three Pt sheet contact pads, which, when put into the measurement chamber, ensured contact with electrical feedthroughs to the electrochemical measurement setup.

During measurement, the sample holder was mounted onto a copper cooling block and held in place by stainless steel shields. The copper block, its tubing, the electrical feedthroughs as well as the tube of the near infrared laser used for heating the sample, were all connected to the same CF 63 flange. The atmosphere was defined by the vacuum pumping system in combination with the mass flow controlled gas inlet near the bottom of the chamber. The water cooled analyser nozzle was moved close to the sample with a distance of 0.5 mm. The sample was then heated directly by the laser through the aforementioned hole in the sample holder.

Technical limitations of the setup restricted the composition of the atmosphere in the NAP-XPS chamber to H_2_ : H_2_O mixtures of approximately 16 : 1 with a total pressure of 0.75 mbar. The effective *p*_O_2__ of the atmosphere and thus the H_2_ : H_2_O ratio was calibrated *via* the chemical capacitance of GDC as shown by Chueh *et al.*^69^ and validated by mass spectrometry (MS) measurements of the residual gas in the differential pumping stage of the NAP-XPS system.

The nozzle diameter of 350 μm is around the same as the X-ray spot size. An Al-K_α_ source with a monochromator achieves a peak width half maximum of 0.6 eV which corresponds to an energy resolution of 0.2 eV. The EIS and *I*–*V* measurements were conducted utilising a Novocontrol Alpha-A High Performance Frequency Analyser (Novocontrol Technologies, Germany) and a Keithley 2611A Source Measure Unit, respectively. The temperature of the sample was determined *via* the YSZ electrolyte resistance.^70^ The electrolyte resistance *R*_YSZ_ was obtained from the high frequency axis intercept in the EIS measurements (*e.g.* see ESI†) by subtracting the resistance of the setup wires (*i.e.* the short circuit resistance at operation temperature). This method yielded a sample temperature of 600(8) °C. The application of an electrochemical DC polarisation in conjunction with simultaneous XPS measurements allowed us to study the formation and electrochemical behaviour of these complex bimetallic catalyst particles where the conventional *ex situ* setup reached its limit.

### 2.4 Electron microscopy

The *in situ* measured samples were further examined *via* scanning electron microscopy (SEM) as well as transmission electron microscopy (TEM) in order to be able to make concrete statements about particle size and especially particle composition (*e.g.* possible alloy formation). For these investigations two types of samples were used – an originally pristine sample, on which the exsolution was first performed within the XPS setup, and another pre-exsolved one. SEM was performed in secondary electron yield mode on both samples employing a FEI Quanta 250 FEG.

The TEM lamella was fabricated *via* standard lift-out techniques using a Thermo Scientific Scios 2 Dual Beam operating with a Ga ion beam at 30 kV accelerating voltage. The lamella was then thinned at an over-tilt angle of ±1° with the same accelerating voltage and subsequently cleaned at angles of ±2° and ±5° at 5 kV and 2 kV, respectively.

TEM images of the prepared lamellas were then recorded on a FEI TECNAI F20 field emission TEM equipped with an X-FEG, operated at 200 kV. High-resolution TEM (HRTEM) and bright field images were obtained with a Gatan RIO 16 CMOS camera and the scanning transmission mode (STEM) images where recorded using a Fischione high-angle annular dark field (HAADF) detector controlled by the Gatan DigiSTEM II system. The particles and perovskite surface were further investigated *via* electron energy loss spectroscopy (EELS) using a Gatan GIF Tridiem spectrometer.

## 3 Results and discussion

Generally, the overpotential at the working electrode *η*_WE_ is calculated by subtracting all other voltage drops across the cell from the set voltage *U*_set_ such as the ohmic drop at the electrolyte and the setup cables (given by the product of the ohmic resistance *R*_ohm_ and the current *I*_DC_) and the non-linear overpotential at the counter electrode *η*_CE_:

3 *η*_WE_ = *U*_set_ − *I*_DC_*R*_ohm_ − *η*_CE_

As a reasonable simplification, the voltage drop at the counter electrode can safely be neglected due to its exceptionally low polarisation resistance, as already described in ref. 66. The electrolyte resistance *R*_YSZ_ was obtained from the impedance spectra, recorded at the beginning and end of each DC experiment, similar to other studies.^60,71–73^

### 3.1 *Ex situ I*–*V* measurements

#### 3.1.1 Activity switching of pure Ni exsolutions

For the first set of *ex situ* experiments we used a pristine sample (*i.e.* freshly deposited and heated for the first time) with the aim of characterising the electrochemical switching behaviour of only Ni immediately after its exsolution.

To maintain the stability of the parent oxide even if the entire amount of Ni is exsolved – which goes hand and hand with a loss of a structure building B-site metal – Ni was not the main constituent of the parent oxide, but only added in relatively low concentration (3% of the B-site cations). To avoid unintentional co-exsolution of also iron, an anodic overpotential was applied based on the calculated thermodynamic switching potentials of Fe^0^/FeO and Ni^0^/NiO for a given mixture of H_2_ : H_2_O. This anodic overpotential – commonly called ‘protection voltage’ throughout this paper – ensured to keep the oxygen chemical potential in the working electrode (*i.e.* the parent perovskite) high enough to prevent Fe from being exsolved, but sufficiently low to allow exsolution of Ni (*e.g.* for H_2_ : H_2_O = 1 : 1 and +10 mV protection voltage – corresponding to +3.1 mV overpotential – the effective oxygen partial pressure in the working electrode 
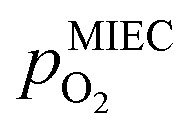
 is 2.3 × 10^−21^ mbar; for further details on the relationship between atmosphere composition and applied overpotential please refer to the ESI† and ref. 65). In addition, a figure in the ESI† describes the considerations as to how we selected the specific protection voltage values for a particular atmosphere.

**Fig. 4:**
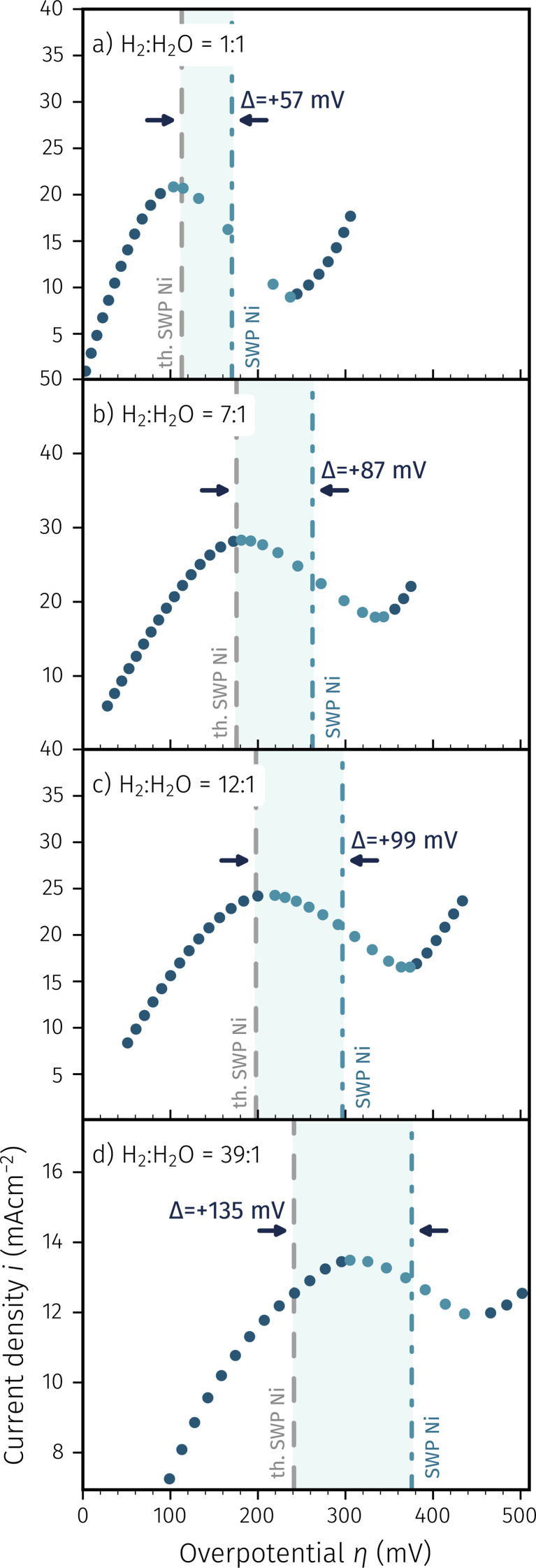
*I*–*V* characteristics of NCFNi working electrodes at different H_2_ : H_2_O mixtures (increasingly reducing conditions from (a) to (d)). The lowest anodic overpotential in each case corresponds to the protection voltage, as the *I*–*V* characteristic was measured starting from this point. The thermodynamically expected switching potential (SWP) is indicated by the grey dashed line whereas highlighted points and the dash-dotted line show the measured SWP. The highlighted region with width *Δ* is the offset between both.

Hence, the electro-catalytic behaviour of pure Ni exsolutions on NCFNi as well as its electrochemical switching behaviour could be studied by recording *I*–*V* curves, or more precisely the branches of *I*–*V* curves with overpotentials being more anodic than the protection voltage. This was done directly after heating up the pristine samples employing the aforementioned procedure. The resulting anodic branches of *I*–*V* curves recorded in different atmospheres ranging from moderately (H_2_ : H_2_O = 1 : 1) to very (H_2_ : H_2_O = 39 : 1) reducing conditions are depicted in Fig. 4.

All *I*–*V* characteristics show the same general appearance with an initial current increase upon increasing polarisation, followed by a current decrease typical for electrochemical activity switching, presumably due to oxidation of nickel (Ni → NiO). To stay consistent with our previous study,^65^ we define the switching potential as the average of the current's local maximum and local minimum. The data points inbetween are highlighted and the resulting switching potentials are shown by the dash-dotted line, whereas the thermodynamically expected switching potential of Ni is depicted as the grey dashed line. The difference between expected and measured switching potential appears to increase with more reducing atmosphere (see highlighted regions and nominal values in Fig. 4). This is in contrast to our previous findings for iron exsolution catalysts, which almost exactly followed the thermodynamic prediction and thus led us to the conclusion that the iron particles were always in thermodynamic equilibrium with their parent oxide.^65^ Moreover, the switching features in the anodic *I*–*V* branches are also much broader in case of Ni than this was the case for Fe.

In this previous study on activity switching of Fe exsolutions, we proposed a model, which aimed to encompass the possible cases of redox switching of exsolution particles based on the different oxygen chemical potential gradients Δ*μ*_O_ of all involved species, namely the particle itself, the parent MIEC oxide and the surrounding atmosphere. These considerations were visualised by plotting the overpotential at which the particle redox (and thus the activity switching) occurs *versus* the effective oxygen partial pressure in the gas phase. In the resulting diagram, different switching situations lead to straight lines with different slopes. An adapted version of this diagram, considering the thermodynamics of the Ni^0^/NiO model system, is depicted in the ESI.† According to these thoughts, during the reduction or oxidation of the particles, two reactions are in competition with each other: (i) electrochemically pumping oxygen between particle and the parent perovskite (compare eqn (4)), and (ii) chemical reaction of the particle with the atmosphere (see eqn (5)). In the extreme case, the state of the particle (metal or oxide) is then governed by the faster of the two reactions, which in the case of iron exsolutions proved to be the electrochemical one. However, if both reactions offer similar reaction rates, a third ‘intermediate’ case can appear where both reactions influence the particle redox. Interpreting the behaviour observed in Fig. 4 in the light of these considerations suggests that the oxygen chemical potential of the nickel particles – in contrast to iron – is indeed affected by both reactions.

4 NiO + 2e^−^ ⇌ Ni + O^2−^

5 NiO + H_2_ ⇌ Ni + H_2_O

The fact that more reducing H_2_ : H_2_O mixtures lead to greater deviations from the thermodynamic switching potential is a strong indication that the Ni^0^/NiO system exhibits this third ‘intermediate’ case. This means that under increasingly reducing conditions, the influence of the atmosphere becomes more pronounced, whereas the electrochemical reaction is losing impact. A process that would explain this behaviour is a transport limitation of oxygen, which occurs in case of the Ni^0^/NiO system, while not being relevant for Fe^0^/FeO exsolutions. For both oxides, NiO and FeO it is a well-known fact that they can exhibit an oxygen excess, which is of the order of 0.01% in case of NiO and about three orders of magnitude higher in case of FeO.^74^ Consequently, it is more accurate to indicate this non-stoichiometry of both oxides by using the notation Ni_1−*x*_O and Fe_1−*y*_O, which we thus use from now on throughout the following text. Since the transport rates of charge carriers through oxides usually depend on the concentration of the respective point defects, it is quite plausible to assume that the transport rate of oxide ions through the highly oxygen-excess material Fe_1−*y*_O is much higher than through the almost stoichiometric Ni_1−*x*_O. Hence, the effect of the atmosphere-dependent switching point together with a rather broad switching feature observed in Fig. 4 can indeed be because of an oxide ion transport limitation through electrochemically formed Ni_1−*x*_O upon anodically oxidising the Ni particles. Due to this additional transport limitation, the total rate of the electrochemical Ni oxidation should be slower as in case of Fe particles, which we propose to cause the observed difference between both particle types. In addition, a natural consequence of this explanation would be a particle size effect, with larger Ni particles being harder to be switched electrochemically. Indeed such a size effect can be observed, as will be shown in more detail below in the discussion of the *in situ* XPS experiments (since additional spectroscopic information as well electron microscopy is very helpful to support our hypothesis see Subsection 3.3 for more information).

#### 3.1.2 Activity switching of bimetallic exsolutions

**Fig. 5:**
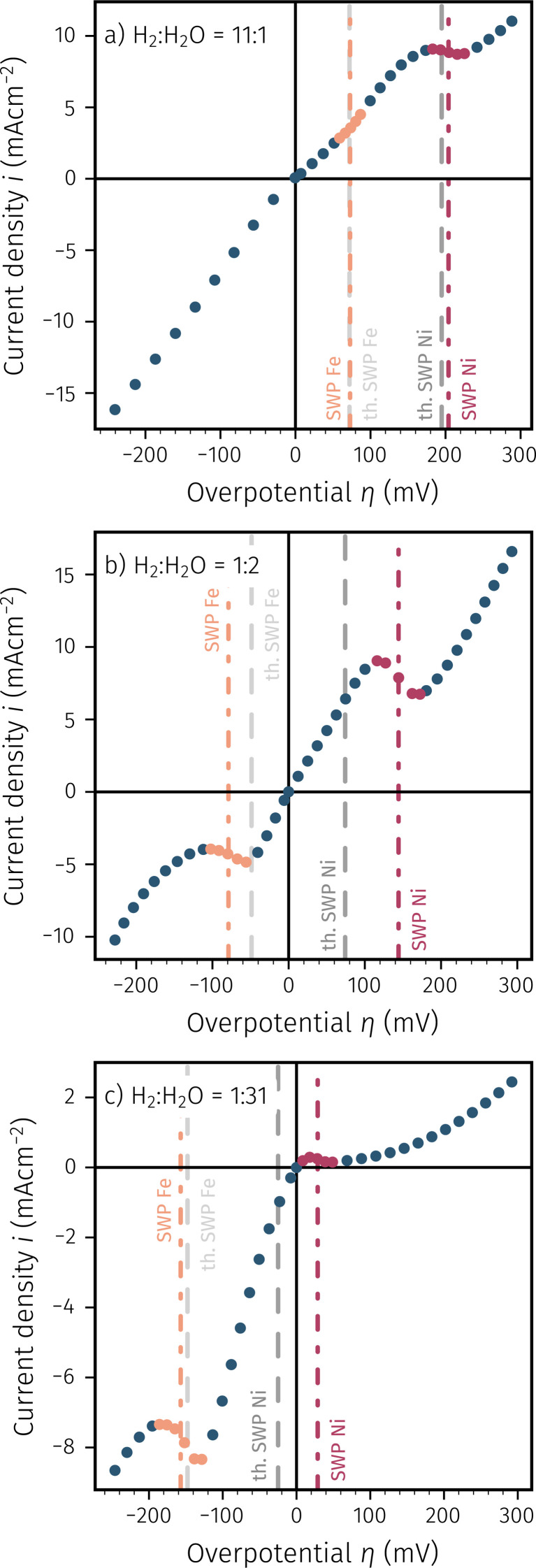
*I*–*V* characteristics of exsolution-decorated NCFNi at different H_2_ : H_2_O mixtures (for increasingly oxidising conditions moving from (a) to (c)): the *I*–*V* curves exhibit two activity switching features, which confirm co-exsolution of Fe^0^ and Ni^0^ particles, as well as their selective switchability. The data points of the switching features are highlighted by different colours – Ni (red) and Fe (orange) – and the nominal switching overpotential is indicated by vertical dash-dotted lines to allow comparisons to their thermodynamically expected values (grey dashed lines).

In the second set of *ex situ* experiments we exposed the samples with only Ni^0^ exsolutions to conditions where also Fe^0^ is thermodynamically favoured to form catalytically active nanoparticles at the electrode surface. This means that after pure nickel exsolution (performed under protection voltage, see above), the working electrode was driven to cathodic overpotentials, which allow iron exsolution. After this, the electrochemical activity switching was studied by again recording *I*–*V* curves (starting from the most cathodic voltage that was used to trigger Fe exsolution). Thus, the resulting curves, in contrast to Fig. 4, now also include the cathodic branch. Examples of resulting *I*–*V* characteristics in increasingly oxidising atmospheres (*i.e.* variations in the H_2_O content) are depicted in Fig. 5. Therein the thermodynamic switching potentials of both transition metals are indicated as grey dashed lines. The experimentally observed switching regions are highlighted and the resulting switching potentials are shown as dash-dotted lines. The more reducing atmosphere in Fig. 5a results in the switching process of both, Fe^0^/Fe_1−*y*_O and Ni^0^/Ni_1−*x*_O, to occur in the anodic branch of the *I*–*V*-curve. Interestingly, for the bimetallic exsolutions the observed iron switching feature in the *I*–*V*-curve is no longer peak-like as this is the case for pure Fe exsolutions.^12,65^ Instead, the Fe^0^/Fe_1−*y*_O transition appears rather like a sudden bend in the *I*–*V* curve towards a steeper slope (compare Fig. 5a). However, changing the atmosphere to more oxidising conditions, thus shifting the switching potential of iron to the cathodic branch, results in a distinct peak-like feature instead (*cf.*Fig. 5b and c), which was also not the case for pure Fe particles. This different appearance of the iron switching feature for bimetallic Ni/Fe particles already indicates that the oxidation of Fe in case of the bimetallic exsolutions, does not lead to an activity decrease as it was described for pure Fe exsolutions.

In accordance with the observations in Fig. 4 and our previous study on iron exsolution,^65^ the deviations from the thermodynamically expected to the measured switching potential are generally much more pronounced for Ni than for Fe. Moreover, upon increasing the water to hydrogen ratio in the gas atmosphere, thus making the atmosphere less reducing, both switching features shift towards the cathodic direction. This can be seen in Fig. 5 when going from panel Fig. 5a*via*Fig. 5b to c, and is both expected and consistent with the activity switching behaviour observed so far.

Another interesting conclusion can be drawn from the direction of both activity switching processes in Fig. 5. The two switching features split the *I*–*V* curves into three distinct parts, which are characterised by three different surface states causing three different electro-catalytic activities. Under strongly cathodic polarisation (*cf.* the very left branch of the *I*–*V* curves), all exsolved particles at the surface are metallic. If the electrode is operated on the middle branch of the *I*–*V* curves (*i.e.* between the two switching features), the iron particles or iron contained in alloy particles is oxidised, leaving just metallic Ni on the surface. Under sufficiently high anodic polarisation (*cf.* the very right branch of the *I*–*V* curves), the Ni^0^ particles are also oxidised leading to an electrode that is decorated only with Ni_1−*x*_O and Fe_1−*y*_O. From the rather low slope of the very right *I*–*V* branch – observable especially for the more oxidising atmospheres – it can be concluded that this entirely oxidic electrode exhibits the lowest electro-catalytic activity. This is understandable, since the loss of metallic particles closes the more active reaction path with H_2_ adsorption/desorption *via* the metal surface^12^ and only the less active pathway *via* the bare perovskite surface remains open.

However, the most interesting phenomenon occurs when switching from strongly reducing conditions (Fe and Ni are metallic) to the middle part of the *I*–*V* curve, where only iron is oxidised, while keeping nickel in the metallic state. This leads to a further increase of the electrode's catalytic activity, which is already more active than in the purely oxidised state. The further activity increase is evident from the fact that upon increasing the anodic/decreasing the cathodic voltage to values right of the Fe^0^/Fe_1−*y*_O switching point, the cathodic current increases abruptly. This is seen in Fig. 5a as a sudden bend in the curve, and in Fig. 5b as well as Fig. 5c as a peak-like switching feature. In all cases, the slope of the middle part of the *I*–*V*-curve is somewhat steeper than the slope of the very left part under strongly reducing conditions.

**Fig. 6:**
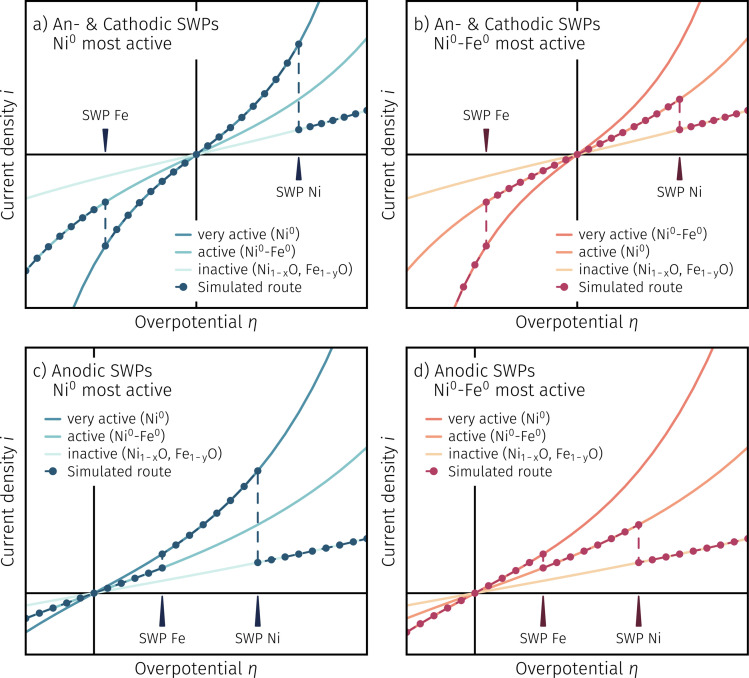
Simulated *I*–*V* curve shapes based on two different criteria: (i) the switching potential locations of both exsolvable constituents ((a) and (b) SWPs in the cathodic and anodic branch; (c) and (d) SWPs exclusively in the anodic branch) and (ii) the most active component ((a) and (c) Ni^0^ or (b) and (d) coexisting Ni^0^ and Fe^0^ metal as the most active components).

To further illustrate this, we simulated the four possible sensible cases in Fig. 6. To do so, we first assume three individual electrochemical kinetics, characterised by three different activity states: very active (highest slope), moderately active (medium slope) and least active (lowest slope) – these are indicated by differently coloured lines. In accordance with the thermodynamics of the system, we define three distinct surface chemical situations: co-existing Ni^0^ and Fe^0^ metal (at overpotentials left of the Fe^0^/Fe_1−*y*_O switching potential), only Ni being metallic (at overpotentials right of the Fe^0^/Fe_1−*y*_O and left of the Ni^0^/Ni_1−*x*_O switching points, respectively), and both metals being oxidised (at overpotentials more anodic than the Ni^0^/Ni_1−*x*_O switching feature). By assigning the activity states to the surface chemical situations, we can simulate *I*–*V* curves exhibiting two switching features. Depending on which activity we assign to which surface chemical situation and taking into account the location of the switching points (*i.e.* whether both switching features appear both in the anodic branch, or iron switches in the cathodic regime while nickel switching still appears at anodic overpotentials), we obtain different directions of activity switching. This means that in some cases the current density jumps either from lower to higher or from higher to lower values. Assuming that fully oxidised nanoparticles always offer the least activity (*cf.* previous work^12,65^ and results above), the number of possible cases is reduced to four, which are depicted in Fig. 6.

Therein, Fig. 6a depicts the case where Ni^0^ is assumed to be the very active and the Ni^0^–Fe^0^ bimetallic exsolutions represent the medium active state with the switching features being in separate branches. As a result two characteristic ‘peak-like’ step changes are visible (both leading to an increase in activity upon reducing the respective driving force), and the steepest slope of the *I*–*V* curve is found in its middle section. In Fig. 6b, for comparison, the bimetallic exsolutions are assumed to offer higher activity compared to the pure Ni^0^ surface decoration. The simulated curve now exhibits just one ‘peak-like’ step change at the switching potential of Ni^0^/Ni_1−*x*_O, whereas the activity regime change from the bimetallic case to pure Ni^0^ appears like a current drop upon decreasing the cathodic overpotential. In addition, Fig. 6c and d depict the cases where the locations of both switching potentials are in the anodic branch. If Ni^0^ is assumed to offer the highest surface activity (see Fig. 6c), the *I*–*V* curve first jumps upwards to the steepest curve upon oxidising iron and under more anodic polarisation experiences a sharp drop in the current due to the oxidation of nickel. Conversely, if the bimetallic exsolutions exhibit the highest surface activity, two distinct peak-like features should be evident in the anodic branch of the *I*–*V* curve (*cf.*Fig. 6d). Comparing the simulated *I*–*V* curves in Fig. 6 with the measured curves in Fig. 5, we can rather straightforwardly conclude that Fig. 6a and c (depending on the working atmosphere) are in agreement with the measured data, even if the measured features do not appear as ideally sharp as in the calculation, but are somewhat blurred. This is probably also the reason why the iron switching point in the anodic *I*–*V*-branch appears more as a bend than as a step to higher currents. We can thus interpret the shape of the *I*–*V* curves in Fig. 5 as a consequence of NCFNi decorated with pure Ni^0^ providing higher electro-catalytic activity than the electrode carrying Ni^0^–Fe^0^ bimetallic exsolutions, and the fully oxidic case being least active.

Often, the origin of different electro-catalytic activities is interpreted by so-called volcano plots. In these diagrams, the rates for water electrolysis/hydrogen oxidation are plotted *versus* the binding energies of the relevant adsorbates. Thereby, it can be observed that the highest reaction rates are typically found at moderate (and thus ‘favourably’ situated) adsorption enthalpies – this is the summit of the volcano. While at lower temperatures platinum group metals are at the top of the volcano curve, the activity of less noble transition metals such as Ni and Fe increases drastically at elevated temperatures (*i.e.* at SOFC/SOEC operation conditions). Theoretical studies show that while pure Ni metal is among the most active elements,^75^ nickel-rich alloys such as Ni_3_Fe could offer even higher activity than pure Ni.^76^ However, the activity of the alloy strongly depends on its actual composition with more than ten mol% of iron already being detrimental to the overall activity.^77^ These findings appear to be in agreement with our observations, as EELS mapping of an exsolved particle with similar pre-history than the ones leading to the behaviour in Fig. 5 suggests only a small portion of it being Ni (*cf.*Fig. 11). The reason for NCFNi decorated with bimetallic exsolution being less active than the case with pure Ni^0^, may thus be the relatively high amount of exsolved Fe^0^. This can be a result of the large amount mismatch between the 3% B-site dopant of Ni and structure element Fe resulting in particles with high iron content. In order to exsolve highly active Ni_3_Fe, it could therefore be an appropriate strategy to integrate the corresponding stoichiometric amounts of the two metals into a non-reducible parent oxide and to completely reduce both exsolvable metals. Pursuing such an approach, however, may be the topic of a forthcoming study, but is beyond the scope of the present paper.

### 3.2 *In situ I*–*V* and XPS measurements

One way to overcome the limitations of *ex situ* electrochemical measurements was the *in situ* coupling of electrochemical experiments with NAP-XPS measurements to observe the oxidation state of exsolved particles upon polarisation. Similar to the *ex situ* experiments we settled on two different types of samples: first, a ‘pristine’ (*i.e.* a freshly PLD grown) working electrode that was heated in the NAP-XPS for the first time, and second, a ‘pre-exsolved’ one which has already experienced *ex situ* polarisation treatment under a protection voltage similar to the first experiment in Subsection 3.1. Hence, the pre-exsolved sample spent a sufficiently long time at elevated temperature under conditions that allow the formation of metallic nickel particles. Due to the longer time under conditions that trigger Ni exsolution, the particles on the pre-exsolved samples are expected to have more time to grow to larger sizes, as also already suggested in literature.^12,21^ Consequently, we have good reason to assume that the two different pre-histories of the two sample types lead to differently sized exsolution particles. This assumption can also be confirmed using SEM and TEM analysis, the corresponding results are discussed in detail below in Subsection 3.3.

As already mentioned above, technical restrictions of the used NAP-XPS setup forced us to set the working atmosphere to H_2_ : H_2_O ≈ 16 : 1 with a total pressure of about 0.75 mbar. (Please note that the reduced total pressure within the NAP-XPS setup may lead to a somewhat slower kinetics of the chemical particle redox reaction (eqn (5)), but would not affect the electrochemical redox reaction (eqn (4)), provided the H_2_ : H_2_O ratio is the same. As a result, the difference between thermodynamically expected and experimentally measured switching point may be slightly smaller in the *in situ* experiments than in the *ex situ* ones.) For *in situ* XPS measurements, both sample types (pristine and pre-exsolved) were heated up also with a protection voltage (see Subsection 3.1), but unfortunately in the NAP-XPS setup the samples are exposed to the reducing atmosphere for a very short time without protection voltage. We therefore assume that the surface of the working electrodes is decorated with exsolution particles that consist mainly of Ni but contain a very little amount of Fe.

**Fig. 7:**
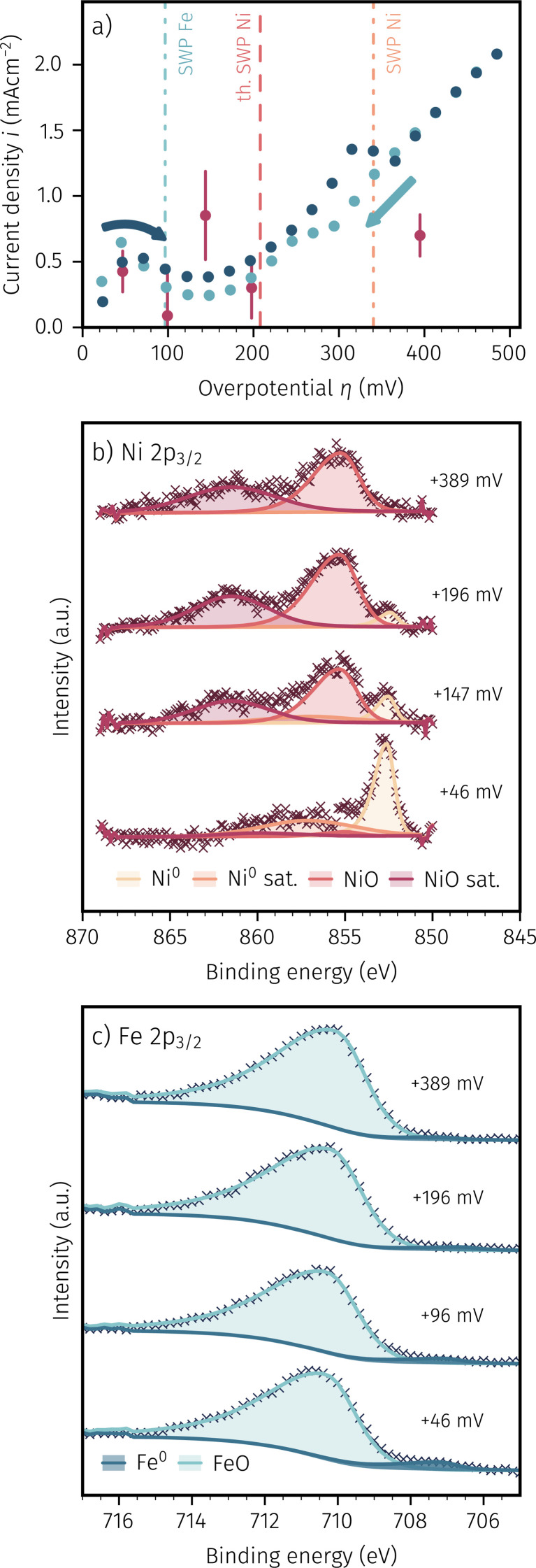
(a) *In situ* recorded *I*–*V* curve of a pristine NCFNi sample in H_2_ : H_2_O ≈ 16 : 1 with arrows indicating measurement directions and coloured lines showing the thermodynamic and measured SWPs for Fe and Ni. XPS spectra of (b) Ni 2p_3/2_ and (c) Fe 2p_3/2_ were recorded at overpotentials indicated by the red coloured points in (a).

While keeping these technical limitations in mind, Fig. 7a depicts the recorded *I*–*V* curves for the ‘pristine’ sample. The differently teal shaded data shows the measurement direction (*i.e.* increasing (dark teal) or decreasing (light teal) anodic polarisation) while the theoretical and measured switching potentials of iron and nickel are given by the dashed and dash-dotted lines, respectively. This *I*–*V* curve was recorded in advance to the spectroscopy measurements to locate points of interest where measuring XPS spectra is expected to offer additional insights. The red data points depict the measured average current and voltage couples recorded during *in situ* XPS measurements at these points of interest. The error bars indicate the change in current density during the comparatively long measurement time required to record the XPS data (compare also the voltages indicated in Fig. 7b and c). The aforementioned unavoidable short-time exposure to the reducing atmosphere in the NAP-XPS chamber may explain the local maxima and minima in the *I*–*V* curves around the thermodynamic switching potential of Fe, and this may also be a reason for the relatively large error bars of the red data points. Nevertheless, even if these working electrodes somewhat differ from their *ex situ* counterparts by small amounts of additionally exsolved iron, in accordance with our *ex situ* results we observed a large deviation of thermodynamically expected and experimentally measured switching potential for Ni^0^/Ni_1−*x*_O.

Fig. 7b depicts the recorded XPS spectra of Ni 2p_3/2_ at the aforementioned points of interest (overpotentials shown on the right hand side of each spectrum). For fitting the spectra, we chose a simple but robust peak model consisting of four mixed Gaussian–Lorentzian peak shapes for the metallic and oxidic component and their respective satellites over an S-shaped Shirley background. Similarly, the Fe 2p_3/2_ spectra in Fig. 7c were fitted with an asymmetric Lorentzian and the same mixed Gaussian–Lorentzian peak shapes for the metallic and oxidic component, respectively. While the absolute numbers from our straightforward peak models may carry some degree of uncertainty, this simplicity serves as its primary advantage. Our primary focus is on discerning the presence of metallic or oxidic species, with less emphasis on precise quantification.

This distinction between nickel and iron particles being in a metallic or oxidic state (possible from Fig. 7b and c) together with the electro-catalytic activity accessible *via* the *I*–*V* curve (Fig. 7a), allows insightful conclusions. For example, the first point of interest at +46 mV – lying even more reducing than the thermodynamic point of iron oxidation – shows a large presence of Ni^0^ as well as a rather small amount of Fe^0^ (the latter also supports our abovementioned suspicion of minimal Fe^0^ exsolution due to the technical limitation of the NAP-XPS setup). Increasing the anodic polarisation resulted in the complete oxidation of iron (*cf.*Fig. 7c at +196 mV and +389 mV), which is in agreement with our previous work on activity switching of iron exsolutions.^65^ In contrast to iron, the anodic oxidation of Ni^0^ particles extends over a rather wide range. At low anodic polarisation the XPS spectra show almost exclusively Ni^0^ (*cf.* +46 mV in Fig. 7b, indicating that virtually the entire amount of Ni from the near surface of the parent oxide was exsolved), whereas NiO appears upon increasing the applied anodic voltage, until the metallic component is completely oxidised (*cf.* +389 mV in Fig. 7b). In general, for nickel, the appearance of a broader switching region is in accordance with the results of the *ex situ* experiments discussed above (Subsection 3.1), and is also evident from the *I*–*V* curve in Fig. 7a as the large deviation between thermodynamically expected and experimentally observed switching point. As discussed in more detail in Subsubsection 3.1.1, we suggest this broadening of electrochemical Ni^0^ oxidation to be related to an oxygen transport limitation in NiO that forms at the interface between Ni particles and NCFNi parent oxide upon sufficient anodic polarisation. As a consequence, one might expect also a particle size dependence of the activity switching of nickel particles, with larger particles being harder to switch – this will be shown in the following.

It is also interesting to note on the pristine sample, that the appearance of an oxidised Ni species already sets in at anodic overpotentials nominally below the thermodynamic transition point of Ni^0^/Ni_1−*x*_O. For example, this may be due to a surface restructuring effect of the perovskite parent oxide that still contains nickel on its B-site, or indeed an oxidation of very small Ni^0^ particles that are less noble and thus easier oxidisable than bulk metal. However, we have to admit that we unfortunately cannot unambiguously explain this effect from the data available so far. To do so, *in situ* TEM may be the method of choice, but due to the large effort involved, such experiments will be the task of a future study.

**Fig. 8:**
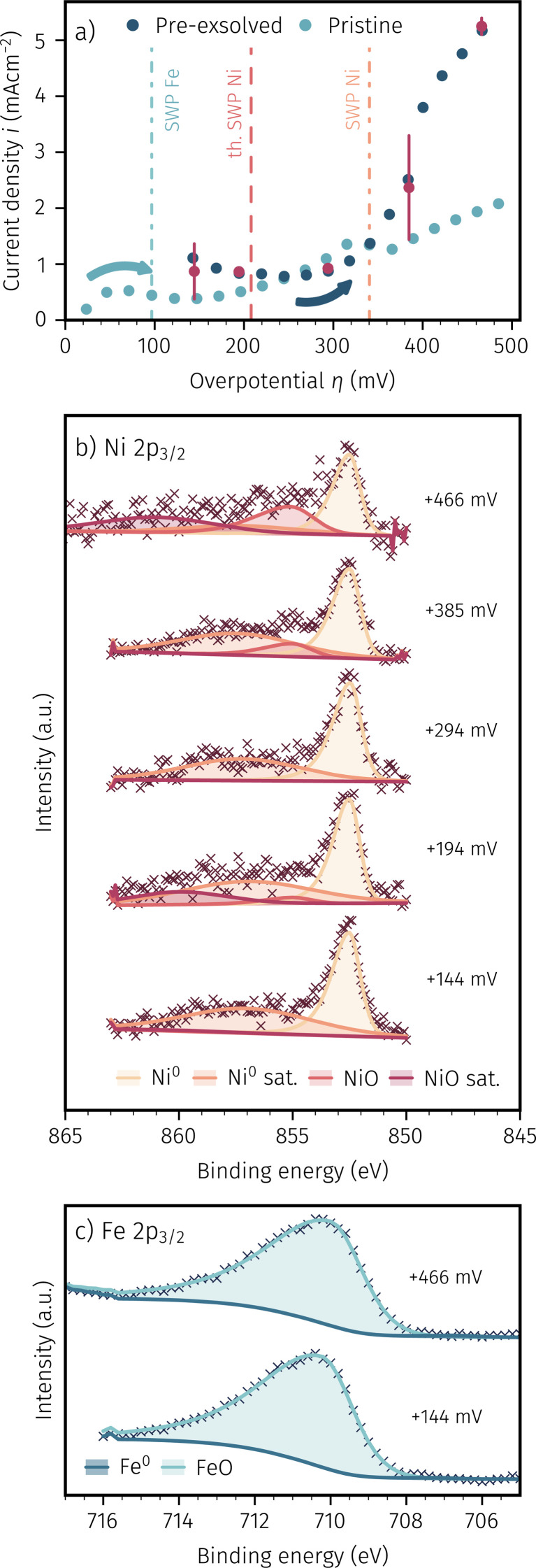
(a) Comparison of the *in situ* recorded *I*–*V* curves of a pre-exsolved and a pristine NCFNi sample in H_2_ : H_2_O ≈ 16 : 1. The *I*–*V* curve of the pristine curve is identical with the dark blue curve in Fig. 7a. The coloured vertical lines indicate the thermodynamic and measured SWPs for Fe and Ni. XPS spectra recorded at the conditions shown with the red data points are depicted in (b) Ni 2p_3/2_ and (c) Fe 2p_3/2_.

A similar *in situ* NAP-XPS experiment as for the pristine working electrode was also conducted for the ‘pre-exsolved’ sample, shown in Fig. 8. Therein, Fig. 8a depicts the *I*–*V* curves of both, the pristine and the pre-exsolved samples. The thermodynamically expected and measured switching potentials are once again shown as dashed and dash-dotted lines, respectively. At anodic overpotentials up to about +340 mV, the electrochemical kinetics of both samples are in acceptable agreement. Above this value, the pristine sample shows a drop in electro-catalytic activity and thus a much shallower course of the *I*–*V* curve. The *I*–*V* characteristics of the pre-exsolved working electrode, however, continues its steepening progression to flatten out somewhat only at very high anodic polarisation. Also, in contrast to the pristine sample, no current decrease in the *I*–*V* curve of the pre-exsolved sample can be observed. This behaviour would imply that no complete oxidation of Ni^0^ and thus no change in surface activity occurred, which would be in accordance with the assumption of large Ni^0^ particles being much harder to oxidise because of a transport limitation of oxygen in a relatively thick Ni_1−*x*_O layer between particle and working electrode. To verify spectroscopically if indeed metallic nickel particles sustain the harsh anodic polarisation, NAP-XPS measurements were conducted at conditions, which are indicated in Fig. 8a with the red data points.

Fig. 8b and c depict the XPS spectra of Ni 2p_3/2_ and Fe 2p_3/2_ for the pre-exsolved sample, respectively. The same fit model as for the pristine sample was used. As expected, Fig. 8c shows that well above the thermodynamically expected switching potential of iron no metallic component could be detected. As already suspected, in the case of Ni^0^ no full oxidation of the active surface particles took place during *ca.* 4 h, even at the highest anodic polarisation (*cf.* XPS spectra at +466 mV). This is insofar remarkable, as the high anodic overvoltage indicates an oxygen activity in the parent oxide that surpasses the effective *p*_O_2__ for Ni oxidation by a significant six orders of magnitude. This permanent presence of the metallic component also explains the lack of a step change and the progression of the *I*–*V* curve of the pre-exsolved working electrode with an continuously increasing slope in Fig. 8a. Furthermore, this finding further corroborates our suggested explanation of an oxygen transport limitation from the parent oxide working electrode to the exsolved Ni^0^ nanoparticles. Consequently, the reducing atmospheric conditions still govern the oxidation state of parts of the nickel particles, which thus stay metallic. As mentioned before, this type of sample has spent a long time at elevated temperatures and reducing conditions, which may have resulted in steady particle growth. Thus, while the portion of the particle close to the perovskite may already be oxidised, the region close to the surrounding atmosphere stays metallic and thus shows high activity for hydrogen oxidation. Indeed, it can be concluded from the Ni 2p_3/2_ spectra, that especially at very high anodic overpotentials an oxidic nickel species appears, which further supports our suggestion of a partial oxidation of the Ni exsolutions.

### 3.3 SEM and TEM analysis

Following the *in situ* experiments, the SEM and TEM images were taken from both pristine and pre-exsolved samples. The aim was on the one hand to determine the average size of the exsolved nanoparticles and on the other hand to measure their composition *via* a combination of scanning TEM and electron energy loss spectroscopy (STEM-EELS). The latter was done to capture details about the spatial arrangement of Fe and Ni within the particles, given that XPS only measures the integral of both metals within the topmost 3 to 5 nm (dictated by the inelastic mean free path of photoelectrons).

**Fig. 9:**
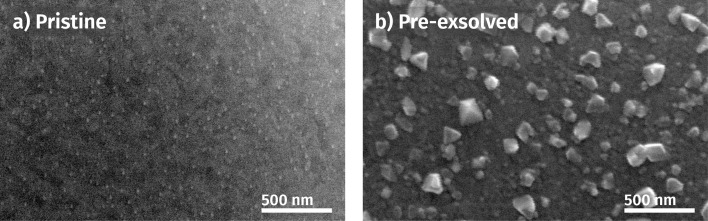
SEM images of (a) pristine and (b) pre-exsolved *in situ* samples with an average 12 nm and 129 nm nanoparticles, respectively.

SEM images of pristine and pre-exsolved samples are shown in Fig. 9. The surface of the pristine sample (*cf.*Fig. 9a) is covered with very small and fine nanoparticles. The average particle size amounts to just 12 nm. In contrast, the pre-exsolved sample in Fig. 9b features comparatively large particles with an average diameter of 129 nm. This exemplifies not only the importance of the samples' pre-history, but also supports our assumption of a particle size effect together with an oxygen transport limitation in Ni_1−*x*_O being responsible for the fact that the Ni^0^ particles on the pre-exsolved samples are not completely oxidised even at very high anodic overpotentials (*cf.*Fig. 8b).

**Fig. 10:**
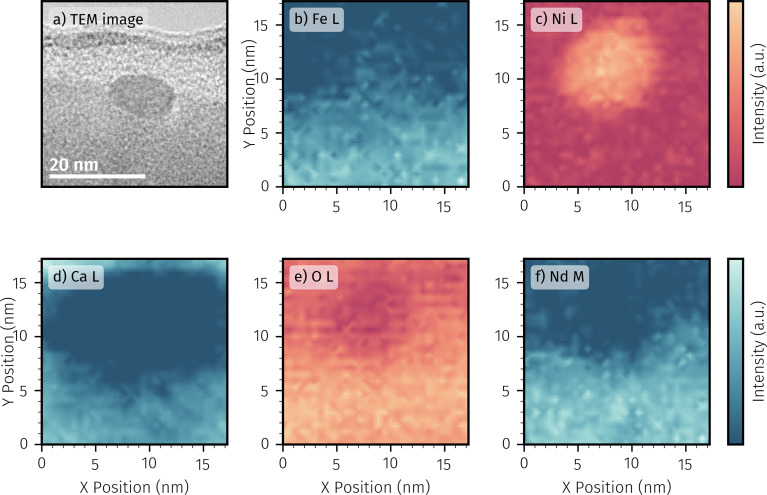
(a) Exemplary TEM image of a surface particle on a pristine *in situ* sample. The bottom part of the image depicts the parent oxide. The light region covering the top half of the particle is a carbon-based protection layer that was required for TEM lamella preparation. STEM-EELS mapping of emission lines of the main perovskite components obtained on another surface nanoparticle: (b) Fe L, (c) Ni L, (d) Ca L, (e) O K, and (f) Nd M.

An exemplary TEM image of a small nanoparticle strongly anchored onto a thin film surface of the pristine sample is depicted in Fig. 10a. STEM-EELS was performed on a similar area also containing a small particle. Fig. 10b to f show the resulting EELS mappings for each main component: Fe, Ni, Ca, O and Nd.

The elemental maps suggest an increase of the Ni intensity at the particle location (*cf.*Fig. 10c). No A-site species (Nd and Ca) appears to be enriched at the particle's site, suggesting the bright protrusions in the SEM image (Fig. 9a) to consist almost exclusively of Ni. Precipitation of A-site elements on the surface can not be detected. The increase in Ca intensity at the very top of the respective elemental map can be attributed to signal overlap between the Ca L line and a signal from carbon in the protection layer. The iron signal is mainly limited to the region of the parent oxide, but also shows a small increase at the nanoparticle location (*cf.*Fig. 10b). This is an indication that the particles are actually an alloy of Ni with minor amounts of Fe. To confirm the validity of this conclusion, EELS line scans were performed on multiple nanoparticle locations. A representative line scan can be found in the ESI.† Moving from the carbon based protection layer to the parent oxide notable amounts of Ni can be detected at the electrode surface between 6 to 24 nm. Quantification of A-site components is either uncertain (Ca due to the aforementioned carbon overlap) or show notable amounts only after particle level (*e.g.* post 24 nm). In case of iron, however, a significant concentration is already present at particle level, supporting the suggested alloy formation to be an effect that is indeed typical for many exsolved particles on the pristine sample. The oxygen signal (Fig. 10e) decreases significantly in the area of the particle, but clearly does not go back to zero, which indicates that it is oxidised. This behaviour is also recognisable in the line scan (*cf.* ESI†). The region of the particle shows a ratio of Ni : O of almost 1 : 1, which suggests that the particle is oxidised to Ni_1−*x*_O.

**Fig. 11:**
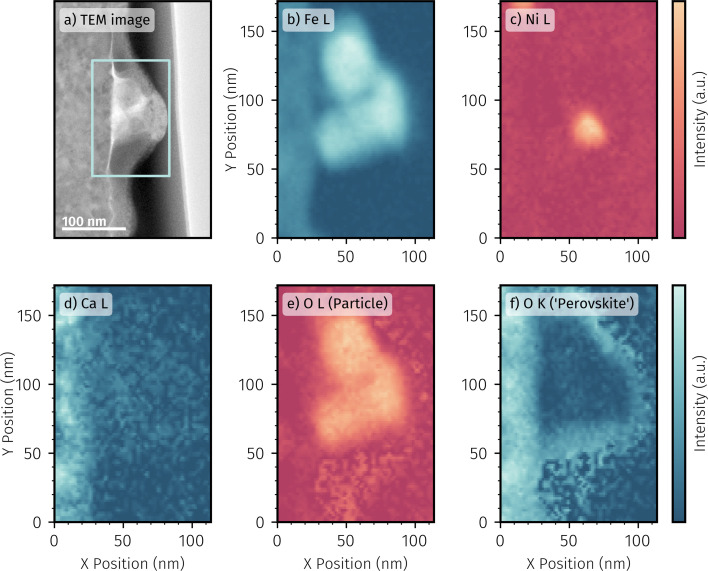
(a) TEM image of a surface particle on a pre-exsolved *in situ* sample. STEM-EELS mapping of main perovskite components in the highlighted area in (a): (b) Fe L, (c) Ni L, (d) Ca L and O K with different chemical environments (e) particle and (f) ‘perovskite’.

By comparison, Fig. 11a depicts the TEM image of a particularly large particle on the surface of the pre-exsolved sample. In contrast to the small particles on the pristine sample, the large particle is much more irregularly shaped and also not so clearly socketed. This could be the result of several electrochemical switching processes carried out on this sample during the *in situ* XPS measurements. EELS elemental maps were recorded on the highlighted area containing the particle and the resulting maps for each element of interest are shown in Fig. 11b to f.

The elemental maps offered multiple insights: even with the relatively large amount of exsolved material, no precipitation of an A site element can be observed. The respective elemental map of Ca is depicted in Fig. 11d; the Nd counterpart is not shown for reasons of limited space (and as it is featureless anyway). A clear difference to the sample with the small particles is the content of Fe and Ni in the large particle and their inhomogeneous distribution in it (compare Fig. 11b and c). The large amount of iron found in the particles is probably due to the fact that only 3% of the perovskite B-site was nickel. After complete exsolution of Ni, only Fe can exsolve further and form metallic particles. This may also be the reason for the inhomogeneity of the large particles. During pre-treatment of these pre-exsolved samples, only nickel particles with little – if any – iron content are formed due to the applied protection voltage. In the large particle in Fig. 11c, this initial exsolution has apparently been preserved as a Ni-rich core. The large amount of iron was then exsolved under sufficiently reducing conditions during the NAP-XPS measurements. The added iron may have partially alloyed into already existing Ni particles or just grown on top of them. The latter is the more likely scenario given the relatively large amount of iron formed in a short time. However, the exact particle growth and evolution process cannot be unambiguously assessed from the post-test TEM analysis. A removal of iron from a large alloy particle under more oxidative conditions may also explain the resulting structure.

In addition to the metals, the oxygen distribution was mapped by means of EELS. In case of the larger particles of the pre-exsolved sample, the oxygen signal's fine structure allows discerning two distinct oxygen species: one primarily situated within the particle and the other coexisting at the parent perovskite thin film's location, as can be seen by comparing the oxygen distribution maps depicted in Fig. 11e (particle) and Fig. 11f (perovskite). This is an indication that the particle is oxidised without rebuilding the perovskite – an observation that is in accordance with the results of an earlier study.^12^

Based on the TEM and EELS results, it can be generally stated that the electrochemical activity switching of bimetallic particles involves a variety of highly interesting processes and dynamics on the atomic level. However, with the *ex situ* investigations that are possible so far, we only see a tiny snapshot of these systems. For example, the elemental distribution in the larger particle, consisting of a smaller Ni-rich core somewhat distanced from the perovskite surface and enclosed by iron oxide, might also be an important factor for potential hindrances to the electrochemical switching of Ni as observed in Fig. 8. However, in order to understand all the details behind bimetallic particle formation and their electrochemical switching, *in situ* TEM measurements would be necessary in any case. These are way beyond the scope of the present study and will be a topic absolutely worth studying in the future.

## 4 Conclusion

In this work, we used a Ni-doped ferrite perovskite material, Nd_0.6_Ca_0.4_Fe_0.97_Ni_0.03_O_3−*δ*_ (NCFNi), as a model system to study the formation process of bimetallic catalyst particles through exsolution. To facilitate a detailed examination of the working electrode's catalytic activity in the H_2_ oxidation/water electrolysis reaction, we prepared dense NCFNi thin films on YSZ single crystal electrolytes with a kinetically fast ceria-based counter electrode. By subjecting the system to a reductive treatment, combining reducing atmospheres and electrochemical polarisation, we achieved the successful generation of highly active Ni^0^ and Fe^0^ nanoparticles on the perovskite surface. Our work highlights the ability to modulate the electro-catalytic capabilities of the exsolution decorated NCFNi electrodes by varying the applied electrochemical polarisation – a phenomenon we term ‘activity switching’. To provide a comprehensive characterisation of the exsolution electrodes, we utilised a combination of electrochemical *I*–*V* measurements, *in situ* NAP-XPS, and electron microscopy.

**Fig. 12:**
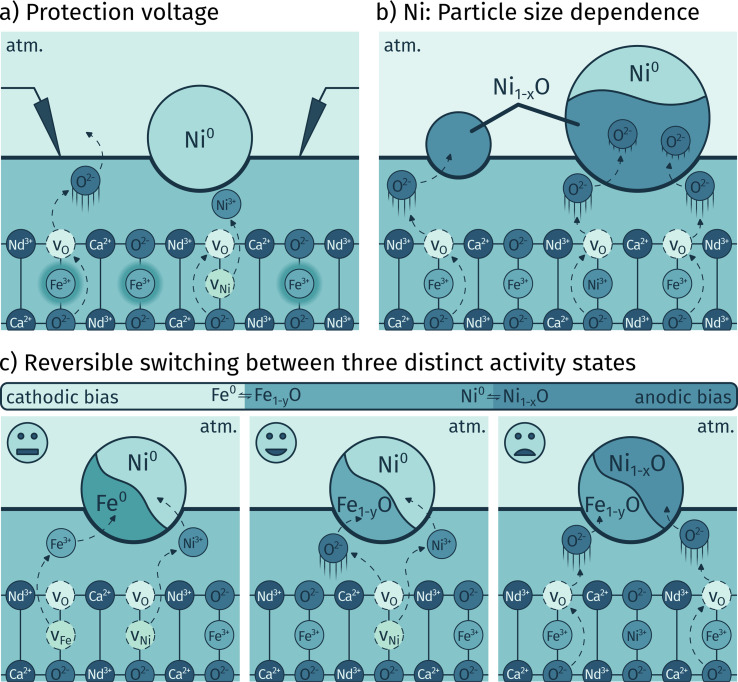
Sketches summarising the results: (a) single exsolution of Ni^0^ in reducing atmosphere and prevention of bimetallic exsolutions by applying a protection bias voltage restraining the less noble iron atoms from leaving the lattice. (b) The behaviour of exsolved Ni nanoparticles depends on the particle size, with an oxygen transport limitation in Ni_1−*x*_O responsible for the observation that larger particles cannot be fully electrochemically switched. (c) Bias controlled switching between three activity states: Fe^0^–Ni^0^ bimetal, solely Ni^0^ and full oxidation exhibiting moderate, high and low catalytic activity, respectively.

To summarise, our experiments revealed the following (which is also sketched in Fig. 12):

• By knowing the exsolution and redox behaviour of the less noble metal – iron in this case – for a given atmosphere, it was possible to exsolve solely the more noble Ni and prevent the formation of bimetallic exsolutions. This was achieved by applying an anodic protection bias voltage counteracting the reductive effect of the hydrogen containing atmosphere (*cf.*Fig. 12a).

• The electrodes decorated solely with exsolved nickel particles revealed a crucial distinction in their behaviour compared to iron exsolutions studied previously.^65^ Both metals principally exhibited an activity switching ability, but the key difference lay in the kinetics of the reactions responsible for the switching behaviour. While in case of iron the electrochemically driven oxidation and reduction of the particles was much faster than the particle redox chemically driven by the atmosphere, the situation for nickel was much more complex. There, for small Ni particles a much slower electrochemical reaction was found, which, however, was able to outperform its chemical counterpart upon sufficiently high anodic polarisation. For larger particles, this was not sufficient either, and the chemical influence of the atmosphere retained the dominant position over the electrochemical polarisation, which also resulted in the loss of the ability for activity switching. We attribute this behaviour to a limitation of oxygen transport through Ni_1−*x*_O that forms at the interface of particle and parent oxide electrode. Ni_1−*x*_O in contrast to Fe_1−*y*_O may exhibit a transport limitation for oxide ions due to its much less pronounced non-stoichiometry, resulting in a slower electrochemical reaction rate compared to iron-based particles (see Fig. 12b).

• This interpretation was also supported by *in situ* NAP-XPS measurements where the larger Ni^0^ particles on pre-exsolved working electrodes could not be oxidised even at very high anodic overpotentials, whereas pristine samples with no prior treatment – and thus much smaller particles – exhibited an electrochemical activity switching ability (*cf.*Fig. 12b).

• Finally, in case of bimetallic Ni^0^–Fe^0^ exsolutions we could show that – as long as they are switchable – they provide the ability to transition back and forth between three distinct activity states by using voltage as the key controlling parameter. These three activity states were identified by the exsolved particles being Fe^0^ and Ni^0^ bimetal, solely Ni^0^, and entirely oxidised. This capability provides a remarkable level of control over the active surface state, which is expected to be highly advantageous for various catalytic applications (see Fig. 12c).

## Data availability

The data supporting this article is available at TU Wien's data repository at https://doi.org/10.48436/rd6gb-aqh43.

## Conflicts of interest

There are no conflicts to declare.

## Supplementary Material

TA-012-D4TA00989D-s001
