# Supplementary material for: Electro-tuned catalysts: voltage-controlled activity selection of bimetallic exsolution particles
Source: J Mater Chem A Mater. 2024 Jul 3;12(31):20386–402. doi: 10.1039/d4ta00989d (PMC11304079; doi:10.1039/d4ta00989d)
Supplement: TA-012-D4TA00989D-s001 [file TA-012-D4TA00989D-s001.pdf]

Electronic Supplementary Material (ESI) for Journal of Materials Chemistry A.
This journal is © The Royal Society of Chemistry 2024

Electro-Tuned Catalysts: Voltage-Controlled Activity Selection of Bimetallic Exsolution Particles

Harald Summerer[1,2], Kirsten Rath[1], Andreas Nenning[1], Thomas Schachinger[3,4], Michael, Stöger-Pollach[3,4], Christoph Rameshan[2,5], and Alexander K. Opitz[1]

[1]TU Wien, Institute of Chemical Technologies and Analytics, Getreidemarkt 9/164-EC, 1060 Vienna, Austria

[2]TU Wien, Institute of Materials Chemistry, Getreidemarkt 9/165-PC, 1060 Vienna, Austria

[3]TU Wien, Institute of Solid State Physics, 1040 Vienna, Austria

[4]TU Wien, University Service Centre for Transmission Electron Microscopy, 1040 Vienna, Austria

[5]Chair of Physical Chemistry, Montanuniversity Leoben, Leoben, 8700, Austria

June 21, 2024

Supporting Information

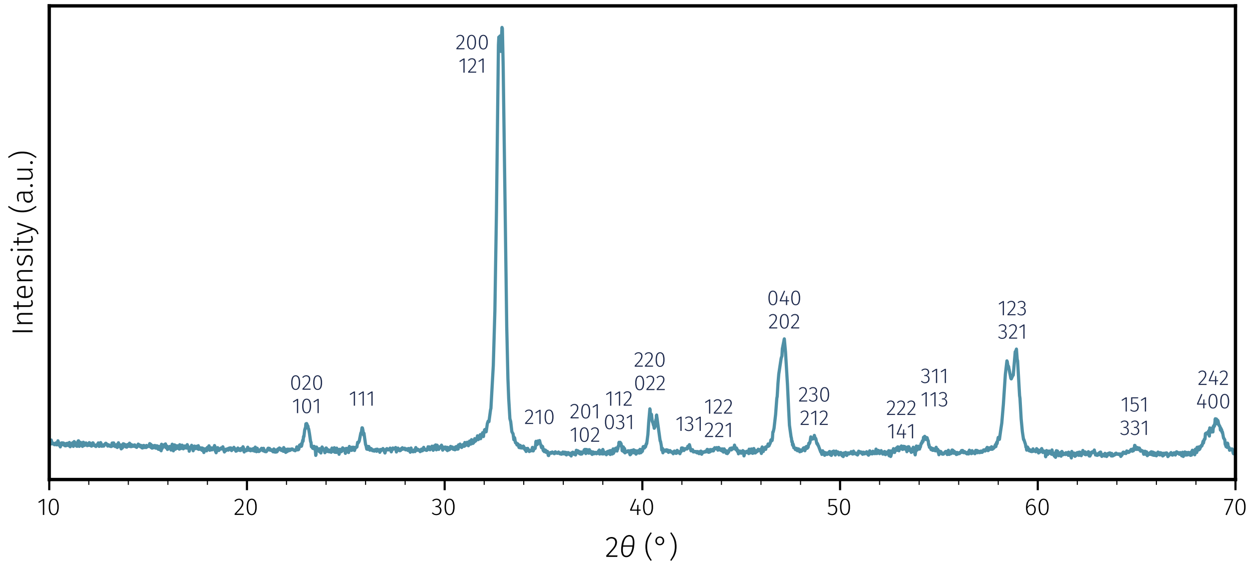

FIGURE S1: XRD pattern of the NCFNi target with (hkl) indices corresponding to orthorhombic, Pnma, space group # 62

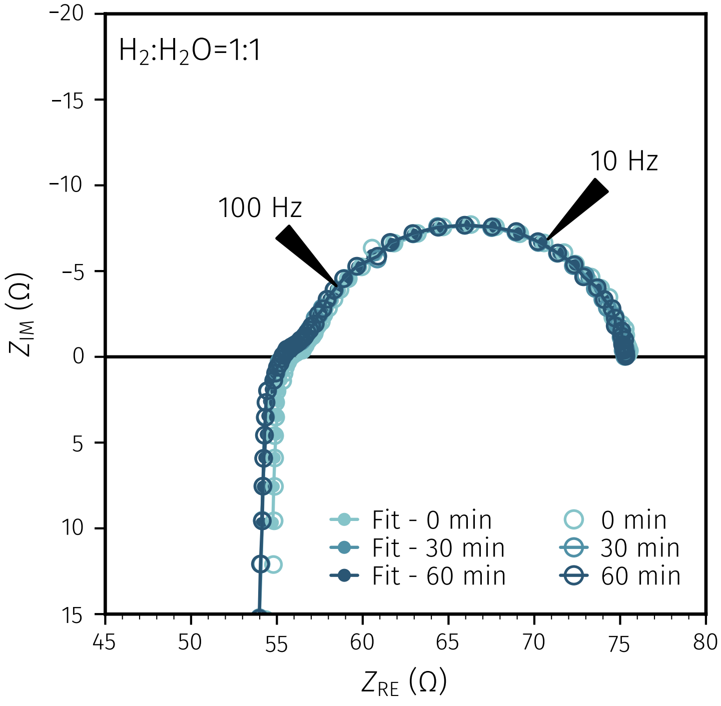

FIGURE S2: Exemplary impedance spectra of NCFNi at 600 °C in H_2:H_2O 1:1 right after heating (0 min), in between (30 min) and at the end 60 min of the 1 h long equilibration period before the start of the DC measurements.

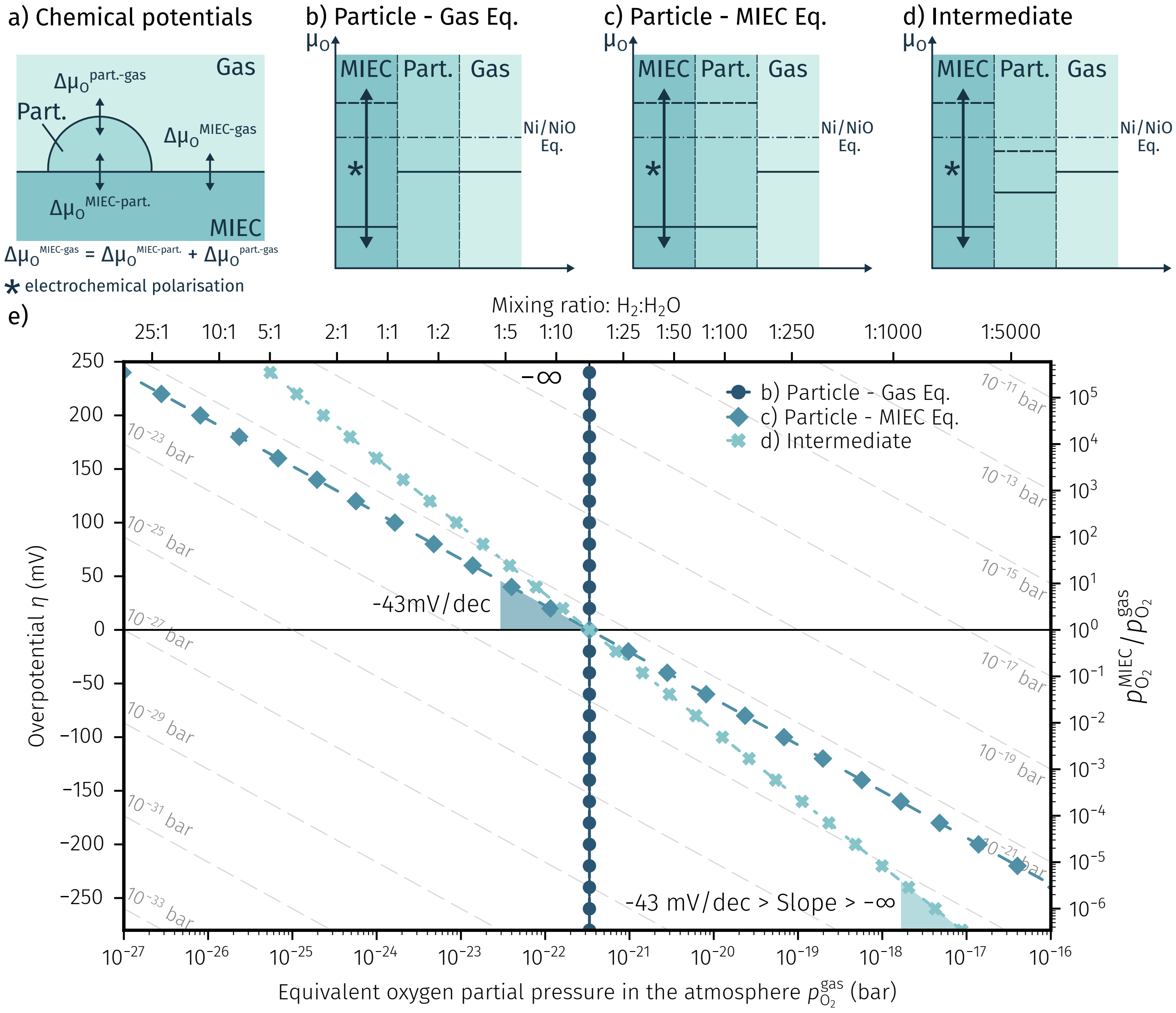

Figure S3: Adapted to Ni^0/NiO from our previous study for Fe^0/FeO[1]: a) Sketch of all gradients in the oxygen chemical potential $\Delta\mu_O$ of the involved phases. b-d) Three different possibilities for the switching behaviour where either b) $\Delta\mu_O^{MIEC-gas} \approx \Delta\mu_O^{MIEC-part.}$ or c) $\Delta\mu_O^{MIEC-gas} \approx \Delta\mu_O^{part.-gas}$ applies as well as an d) intermediate one with both, MIEC and gas, affecting the particle. e) Resulting Ni/NiO equilibria for all three cases shown in a semilogarithmic plot of the overpotential η at the working electrode versus the equivalent oxygen partial pressure $p_{O_2}^{gas}$ in the atmosphere. The dashed lines represent the isobars of $p_{O_2}^{MIEC}$.

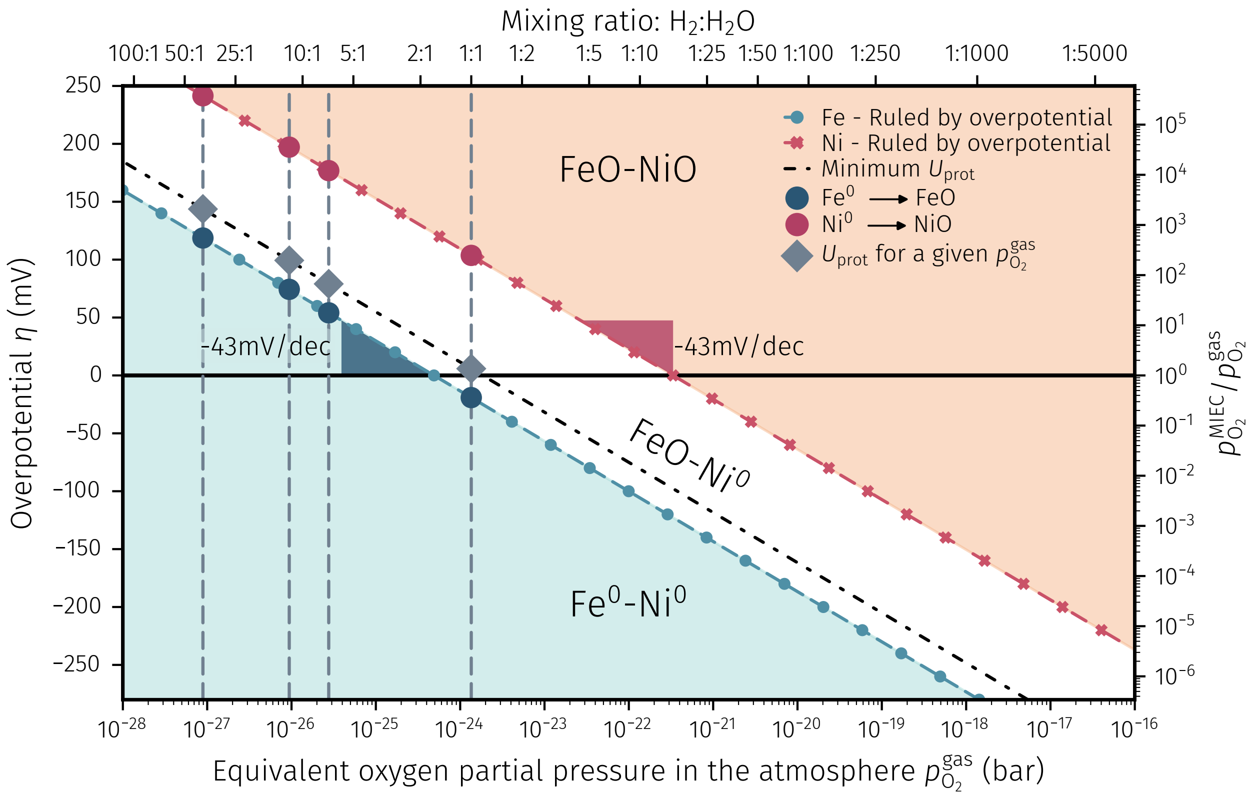

Figure S4: Semilogarithmic of the overpotential η at the working electrode versus the equivalent oxygen partial pressure $p_{O_2}^{gas}$ in the atmosphere. If the oxidation state of exsolved iron and nickel particles is mainly governed by the electrochemical reaction, it is possible to distinguish between three distinct segments: Fe^0—Ni^0 (light teal), FeO—Ni^0 (white) and FeO—NiO (orange). The corresponding working atmospheres in Fig. 4 are depicted as grey dashed lines and the dark teal/red dots mark the overpotential values where iron/nickel metal transition to their respective oxides. The protection voltage U_{prot} for a given atmosphere (grey diamonds) was chosen by shifting the Fe^0—FeO equilibrium line 25 mV up (dot-dashed black line) and thus preventing Fe^0 from ever being the more stable surface state.

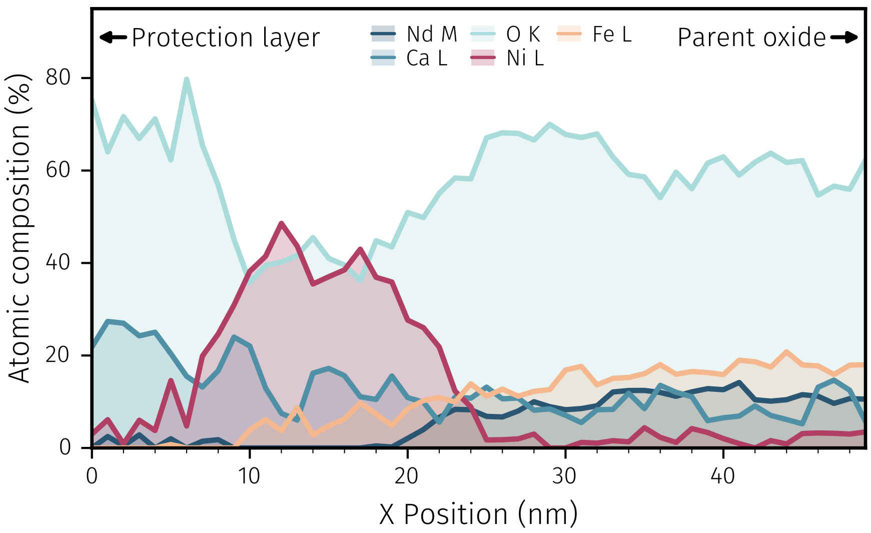

Figure S5: Atomic compositions of all main components (Nd, O, Ca, Ni, Fe) as determined by an EELS line scan of a pristine *in situ* sample ranging from the protective layer through the surface particle to the parent oxide. A clear accumulation of Ni between 6 nm and 24 nm is recognisable.

References

(1) H. Summerer, A. Nenning, C. Rameshan and A. K. Opitz, *EES Catalysis*, 2023, **1**, 274–289.
